# *Histoplasma* stomatitis unveiled: Not all opportunistic infections get better after initiation of antiretroviral therapy

**DOI:** 10.1002/ccr3.3799

**Published:** 2021-01-19

**Authors:** Saurabh Bansal, Namrata Singhania, Chandra Mouli Nukala, Anil Kumar Singh, Laith Al‐Rabadi

**Affiliations:** ^1^ Department of Internal Medicine University of Illinois College of Medicine at Peoria Peoria IL USA; ^2^ Department of Hospital Medicine Mount Carmel East Hospital Columbus OH USA; ^3^ Department of Hospital Medicine CHI St Vincent Infirmary Little Rock AR USA; ^4^ Department of Internal Medicine Geisinger Community Medical Center Scranton PA USA; ^5^ Division of Nephrology University of Utah Salt Lake City, Utah USA

**Keywords:** antiretroviral therapy, *Histoplasma*, HIV, IRIS, stomatitis

## Abstract

Immune reconstitution inflammatory syndrome in AIDS patients can lead to an initial worsening of underlying diseases due to body's ability to mount a strong immune response after recovery of CD4 counts.

## Graphical abstract

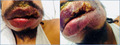

## 1 INTRODUCTION

Histoplasmosis occurs primarily in the lungs but can disseminate in immunocompromised patients. Serology can be negative in patients with local infection, making the diagnosis challenging. Definite diagnosis is by microscopic examination of the tissue sample. We report a rare case of Histoplasma stomatitis whose lesions manifested after initiating antiretroviral therapy.

*Histoplasma* capsulatum is a dimorphic, round, budding yeast that is found primarily along the Mississippi and Ohio River valleys in the United States but can be seen in other areas also.^1^ Infection occurs by inhalation of conidia, which primarily manifests initially as pulmonary infection, but via hematogenous route, dissemination can occur, especially in high‐risk immunocompromised patients.^1^ Dissemination occurs in extrapulmonary organs causing gastrointestinal infection, stomatitis, mucositis, central nervous system, or bone marrow infection.^2^ Immune reconstitution inflammatory syndrome (IRIS) is a phenomenon in which there is paradoxical worsening of pre‐existing disease or unmasking of subclinical disease after starting antiretroviral therapy (ART) due to improvement in immune response of the body.^3^, ^4^ Mucocutaneous lesions due to H. capsulatum have been rarely associated with IRIS.^5^, ^6^, ^7^ We herein report a case of H. capsulatum stomatitis in a HIV‐infected patient after resumption of his ART.

## 2 CASE PRESENTATION

**FIGURE 1:**
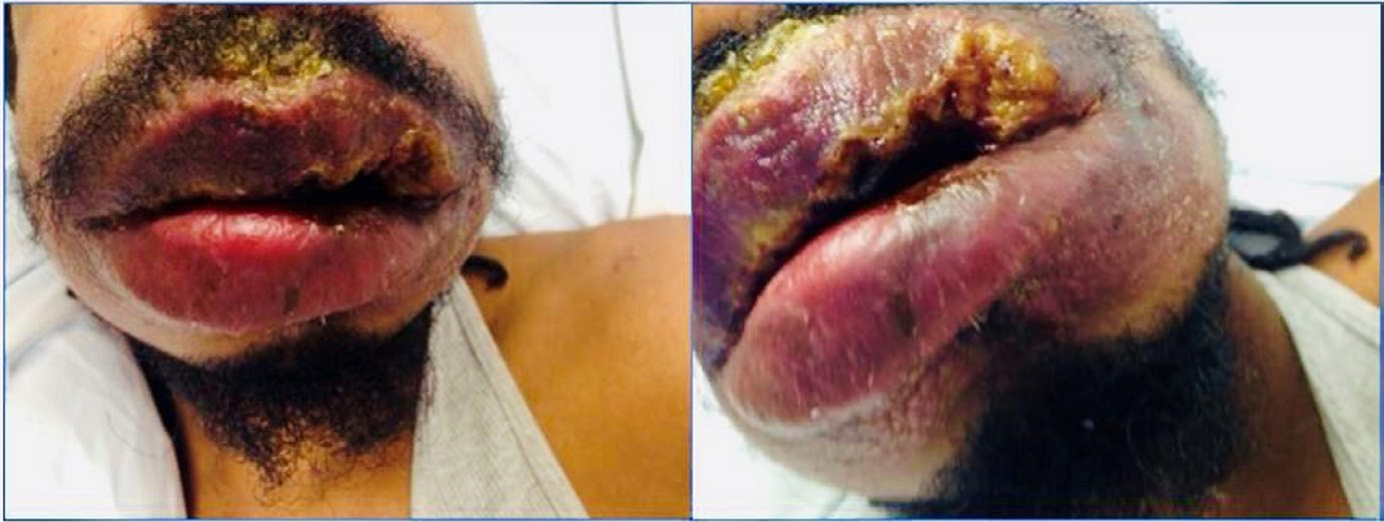
*Histoplasma*‐associated stomatitis showing swollen lips and superficial ulcers with heaped‐up margins

A 34‐year‐old African American man with a past medical history of hemophilia A requiring twice‐weekly recombinant factor VIII infusions, HIV‐1, and hepatitis C infection presented with 3‐month history of intermittent fever, significant weight loss, and worsening lip swelling and ulceration. He became compliant with ART 6 months ago as evident by improvement in CD4 count from 94 cells/mm^3^ to 160 cells/mm^3^. His mucocutaneous lesions appeared two months after improvement in CD4 count. He received multiple antibiotics and steroids in the past with no relief. He was on acyclovir 400 mg twice a day and dapsone 100 mg once daily for herpes simplex virus (HSV) and Pneumocystis jiroveci prophylaxis for the past two years, respectively. Physical examination revealed significantly swollen lips and multiple superficial ulcers with heaped‐up margins (Figure 1). No obvious hepatomegaly or splenomegaly was noted. Differential diagnosis included herpetic ulcers, bacterial infections, fungal infections, or angioedema. He was initially started on valacyclovir for presumed HSV infection. HSV polymerase chain reaction taken from the base of lip ulcer was negative. HIV and hepatitis C viral loads were undetectable. Complement C1 esterase inhibitor level was negative. Gram stain, acid‐fast stain, mucicarmine stain, fungal blood cultures, and urine and serum *Histoplasma* antigen were negative. Complement fixation test was positive for H. capsulatum mycelial and yeast forms (titer > 1:256). Serum immunodiffusion assay was strongly positive for H. capsulatum “H” and “M” bands but negative for Aspergillus fumigatus, *Blastomyces dermatiditis,* and *Coccidiodes immitis*. Biopsy of the lips revealed granulomatous and mixed inflammatory infiltrate predominantly with lymphocytes with areas of necrosis and presence of small budding yeast forms. The patient was diagnosed with H. capsulatum–associated stomatitis and was started on itraconazole 200 mg twice a day for total of twelve months. He demonstrated dramatic regression of oral edema and ulcerations at six‐month follow‐up visit.

## 3 DISCUSSION

Histoplasmosis is the most common endemic mycosis in AIDS patients, frequently observed in Mississippi and Ohio River valley.^1^ Incidence is reportedly 3.4 cases per 100,000 person‐years in the United States.^1^ It usually manifests as primary pulmonary infection from inhalation of microconidia but can disseminate hematogenously in immunocompromised patients—leading to skin, neurological, bone marrow, or gastrointestinal abnormalities.^2^ The most common symptoms of pulmonary histoplasmosis are fever, chills, weakness, and cough with chest radiograph commonly revealing interstitial or reticulonodular infiltrates.^2^ Other findings seen on chest imaging are pneumonia‐like consolidation with or without hilar lymphadenopathy, and/or cavitary lung lesion.^2^ Disseminated histoplasmosis patients often have progressive weight loss and fever with other clinical symptoms depending on the organ system involved.^8^

Diagnosing histoplasmosis in mild‐to‐moderate local infection can be challenging. Serum and urine antigen testing and tissue cultures may remain negative.^8^ Definite diagnosis is achieved by microscopic examination of tissue sample, which can reveal immature forms.^9^ Both the immunodiffusion test and the complement fixation test should be used for workup. Antibody detection of “H” and “M” bands through immunodiffusion has higher sensitivity than complement fixation.^8^ Titers > 1:32 or increasing titers when checked at an interval of 1‐2 weeks are highly suggestive of H. capsulatum infection.^9^, ^10^ Antibody testing can have low sensitivity in immunocompromised patients.^10^ New emerging tests such as microbial cell‐free DNA testing can have quick turnaround time and help in diagnosing the disease sooner.^11^, ^12^

Unveiling of manifestations of histoplasmosis as a result of immune reconstitution inflammatory syndrome (IRIS) has been rarely reported in the literature.^5^, ^6^, ^7^ Patients with active HIV infection who are not taking ART and have low CD4 count and high viral load have poor initial immune response to opportunistic infections.^13^ Rapid improvement in immune function and suppression of viral load after initiation of ART can lead to systemic or local inflammatory response at sites of pre‐existing infection, which were not clinically apparent before initiation of ART. Increases in T lymphocytes after initiation of ART activate suppressed immune response leading to inflammation and granuloma formation. This can happen in 1 to 3 months after initiation of ART.^14^ This phenomenon is more commonly reported in patients with tuberculosis; therefore, tuberculosis remains the main differential in suspected IRIS patients.

Our patient developed his lesions two months after he started taking ART as evident from his improvement in CD4 counts and suppression of HIV viral load. Strong immune response to localized H. capsulatum infection as evidenced by both immunodiffusion and complement fixation assays is also compelling. Treatment of histoplasmosis depends on clinical form and severity and involves various antifungals such as azole drugs in mild‐to‐moderate disease or amphotericin B in moderate‐to‐severe cases.^15^ Also, immunocompromised patients with severe disease at presentation are frequently treated with amphotericin B. In patients with severe disease, after initial induction phase of one to two weeks with amphotericin B, treatment can be switched to oral azole drugs such as itraconazole. The preferred duration for mild‐to‐moderate infection is 12 months and longer for severe disease, keeping in mind, lifelong therapy may be needed in selected individuals.^16^ Monitoring of drug levels is recommended to ascertain compliance and adequate levels. *Histoplasma* antigen levels are frequently used to monitor treatment response. For the treatment of IRIS, the use of glucocorticoids, which may seem counterintuitive in an immunocompromised patient with disseminated infection, may be needed in selected individuals.

In summary, H. capsulatum–associated stomatitis can be diagnosed by microscopic examination of tissue sample along with immunodiffusion and complement fixation tests. Antigen test can be negative in localized infection. It can be associated with IRIS with paradoxical worsening of mucocutaneous lesions after initiation of ART.

## CONFLICT OF INTEREST

None declared.

## AUTHOR CONTRIBUTIONS

SB and NS: contributed equally in preparing the manuscript and reviewed the literature. CMN, AKS, and LA: critically revised the manuscript.

## ETHICAL APPROVAL

Ethics committee was not consulted for approval as it is a case report, and all possible efforts were made to maintain complete anonymity.

## Data Availability

The authors confirm that the data supporting the findings of this study are available within the article and no additional source data are required.
